# Neural evidence for progressive decline of visual face recognition ability in Alzheimer’s disease

**DOI:** 10.1093/braincomms/fcag390

**Published:** 2026-10-22

**Authors:** Justine David, Lisa Quenon, Adrian Ivanoiu, Laurent Koessler, Lise Colmant, Yasmine Salman, Lara Huyghe, Bernard Hanseeuw, Bruno Rossion

**Affiliations:** INTERPSY, Université de Lorraine, Nancy F-54000, France; Institute of Neuroscience (IONS), Université Catholique de Louvain (UCLouvain), Brussels B-1200, Belgium; Neurology Department, Cliniques Universitaires Saint-Luc, Brussels B-1200, Belgium; Institute of Neuroscience (IONS), Université Catholique de Louvain (UCLouvain), Brussels B-1200, Belgium; Neurology Department, Cliniques Universitaires Saint-Luc, Brussels B-1200, Belgium; IMoPA (UMR7365), Université de Lorraine, CNRS, Nancy F-54000, France; Institute of Neuroscience (IONS), Université Catholique de Louvain (UCLouvain), Brussels B-1200, Belgium; Neurology Department, Cliniques Universitaires Saint-Luc, Brussels B-1200, Belgium; Institute of Neuroscience (IONS), Université Catholique de Louvain (UCLouvain), Brussels B-1200, Belgium; Institute of Neuroscience (IONS), Université Catholique de Louvain (UCLouvain), Brussels B-1200, Belgium; Institute of Neuroscience (IONS), Université Catholique de Louvain (UCLouvain), Brussels B-1200, Belgium; Neurology Department, Cliniques Universitaires Saint-Luc, Brussels B-1200, Belgium; Institut des Sciences Cognitives-Marc Jeannerod (UMR5229), CNRS, Université Claude Bernard Lyon 1, Bron F-69500, France; Service de Neurologie, Université de Lorraine, CHRU-Nancy, Nancy F-54000, France

**Keywords:** Alzheimer’s disease, face recognition, EEG, fast periodic visual stimulation

## Abstract

Alzheimer’s disease (AD) is associated with deficits in recognizing familiar faces already at prodromal stages due to significant episodic and semantic memory impairment. However, whether the ability to visually discriminate and match unfamiliar faces for their identity also declines in AD remains unclear. We addressed this issue with a robust implicit electroencephalographic (EEG) measure of face identity recognition (FIR) relying on fast periodic visual stimulation (FPVS). Thirty cognitively unimpaired (CU) individuals and thirty AD patients, including seventeen patients with mild cognitive impairment (MCI) due to AD and thirteen patients with dementia due to AD viewed stimulation sequences of the same unfamiliar face identity repeated at 6 Hz across size changes, with other identities appearing every 5 faces, eliciting a neural 1.2 Hz response indexing FIR. Stimuli were also presented upside down to control for low-level visual effects. While CU individuals exhibited robust FIR EEG responses at 1.2 Hz (and harmonics) over the occipito-temporal cortex, larger for upright than inverted faces, this response and the inversion effect were significantly reduced in patients with dementia due to AD but preserved in patients with MCI due to AD. These results indicate that unfamiliar FIR is preserved in patients at the prodromal stage, suggesting that familiar FIR difficulties previously observed during the prodromal phase of AD may stem from other cognitive factors, such as long-term memory impairments. In contrast, deficits in discriminating unfamiliar faces seem to emerge as the disease progresses. Overall, this study provides novel evidence for the processes underlying face identity recognition deficits in AD and highlights the diagnosticity of FPVS-EEG for assessing specific cognitive processes in neurodegenerative populations.

## Teaser

David *et al*. showed that Alzheimer's patients in early stages can still discriminate unfamiliar faces’ identity, while in advanced stages, this ability is impaired. Using brain recordings, they revealed that early-stage patients’ familiar face recognition difficulties do not stem from perception deficits.

## Graphical Abstract

**Graphical Abstract:**
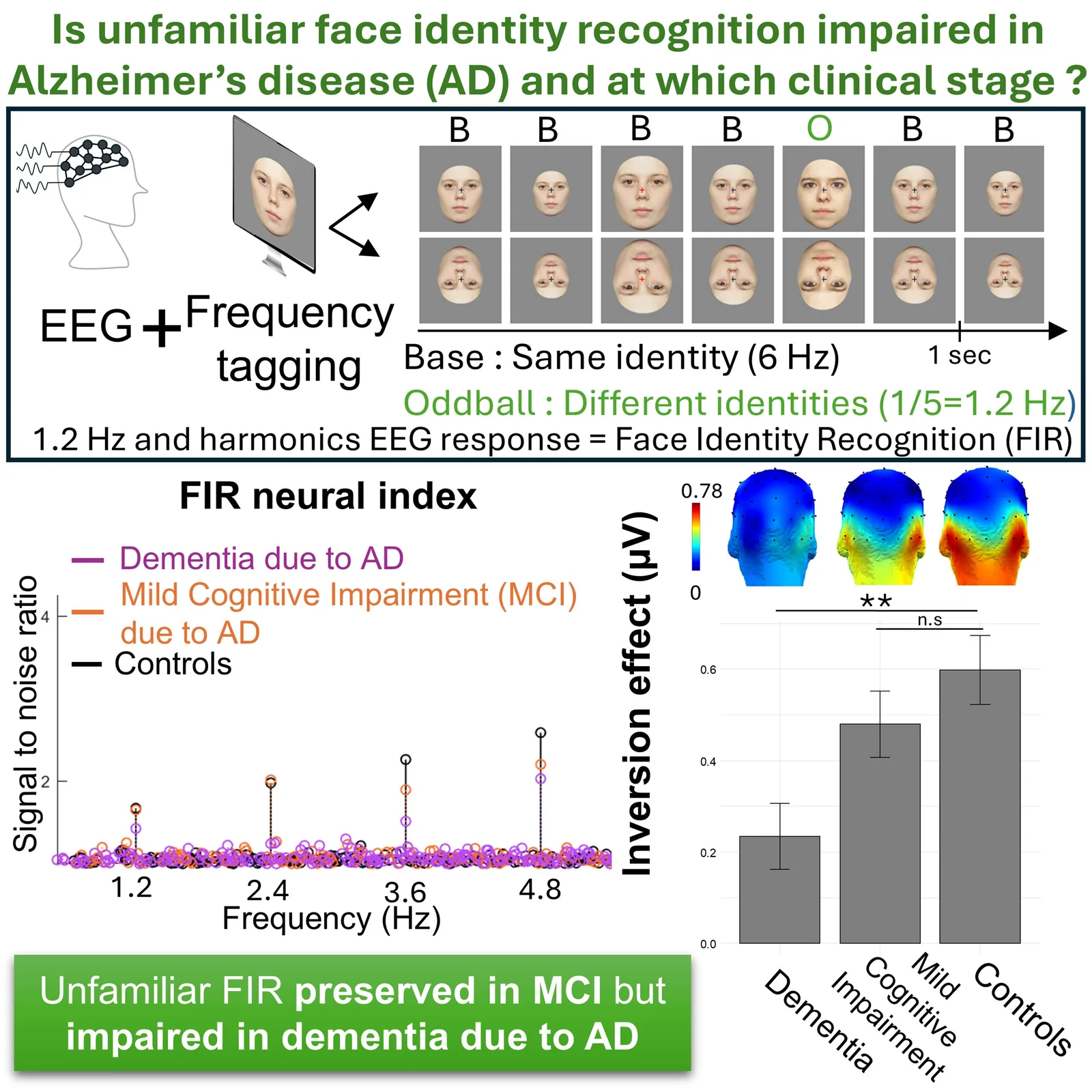

## Introduction

Patients with Alzheimer’s Disease (AD) often fail to recognize familiar face identities,^1^ which can be emotionally painful for patients’ relatives, friends, and acquaintances. Since face identity recognition (FIR) is fundamental for social interactions, this impairment may progressively disrupt social functioning and promote social isolation, accelerating cognitive and functional decline.^2,3^ In light of the rising prevalence of AD,^4^ a clearer understanding of the cognitive mechanisms underlying this FIR deficit is therefore essential for developing effective support, intervention, and compensatory strategies that may improve patients’ quality of life and potentially slow cognitive deterioration.

FIR impairment has been mainly demonstrated with explicit behavioural recognition of recently learned pictures of unfamiliar faces^5-10^ or famous faces.^11-17^ Deficits in recognizing previously encoded face identities have been described as early as the prodromal stage of the disease^9,18,19^ (but see^8^) defined by the presence of disease-related biological markers and mild cognitive impairment (MCI), despite preserved daily functioning.^20^

However, the specific cognitive processes underlying the FIR impairment in AD remain unknown. While it has primarily been attributed to semantic and episodic memory deficits affecting the encoding and retrieval of information in long-term memory,^1,18^ it has also been interpreted as reflecting impaired visual processes.^9,21^ The present study thoroughly investigates this latter hypothesis by evaluating whether the core ability to discriminate and match pictures of unfamiliar facial identities, devoid of semantic and verbal associations, is truly affected in AD and, if so, at which stage of the disease.

Unfamiliar FIR^22^ is typically evaluated with pictures of unfamiliar faces to discriminate or match for their identity (e.g. *Benton Facial Recognition Test*^23^; Cambridge Face Memory Test^24^). While the most cognitively impaired AD patients are clearly impaired at unfamiliar face identity matching tasks,^25-27^ studies with mild AD dementia patients found more nuanced results, with relatively well-preserved performance for the majority of patients^28,29^ or impairment under specific conditions only (depending on face size,^30^; changes in head orientation^31^). Importantly, these latter studies have investigated unfamiliar FIR ability only during the dementia stage of the disease, leaving the prodromal stage largely unexplored. To our knowledge, only two studies investigated unfamiliar FIR performance in MCI due to AD patients, demonstrating their significantly impaired performance at unfamiliar face discrimination with changes in spatial configuration or colour of mouth and eyes,^32^ and on a typical unfamiliar face matching task.^10^ These results suggest that deficits in discriminating/matching faces based on visual cues only would arise already at the prodromal stage of AD. However, since these studies included only patients with MCI due to AD, it cannot be determined whether this deficit is less pronounced than in the dementia stage. Moreover, as for all the aforementioned studies, interpretation of the results is limited by the use of behavioural tasks in which participants are explicitly asked to determine whether faces belong to the same identity or not, instructions that can lead to deficits due to other cognitive impairments (i.e. requirement to understand and memorize instructions, decision-making processes, selective/spatial attention to one of multiple faces concurrently presented, explicit selection and motor planning for behavioural output, etc.). Finally, while efficient neurotypical (familiar or unfamiliar) FIR requires 1 to 2 gaze fixations at most,^33-35^ behavioural studies present face stimuli for several seconds or even unlimited times to patients, encouraging atypical variable gaze explorations and use of compensatory mechanisms. For all these reasons, whether AD patients’ ability to recognize the identity of (unfamiliar) faces based on visual cues only is impaired, and if so to what extent and at which stage of the disease, remains unclear.

Taking these issues into consideration, here we implicitly measured unfamiliar FIR in AD with a sensitive and objective electroencephalographic (EEG) approach in which neural activity is expressed and quantified at stimulation frequencies (‘steady-state evoked potentials’, SSVEP^36^;^37,38^ for reviews). Specifically, we used a face identity ‘oddball’ paradigm extensively validated (>35 published studies since^39^; see^40^ for review; more recent studies: e.g.^41,42^). In this paradigm, an unfamiliar face identity photograph without external features is repeated at a rate of 6 Hz (i.e. 6 images per second), allowing only a single gaze fixation per face, with substantial variation in image size across repetitions. Every five images, a different unfamiliar face identity appears (i.e. AAAABAAAACAAAAD…; Fig. 1). This periodic change in face identity elicits an electrophysiological response precisely at the identity change frequency (i.e. 6/5 = 1.2 Hz), predominant over right occipito-temporal regions.^39,40^ As for other fast periodic visual stimulation (FPVS) paradigms successfully used in AD patients in recent years,^43,44^ this oddball-like paradigm is associated with significant advantages: an implicit neural response measured objectively (i.e. at pre-defined frequencies of interest) and a high sensitivity [i.e. high Signal-to-Noise Ratio (SNR)].^37,38^ The validity of this approach is well established: response amplitude is markedly reduced when the same face stimuli are presented upside-down or contrast-reversed (two manipulations known to affect human FIR),^39^ abolished or severely reduced in individuals with prosopagnosia,^45-47^ and is maximal in typical face-selective regions within the ventral occipito-temporal cortex (VOTC) in intracerebral recordings.^48^ Hence, this neural measure is particularly well-suited to assess the integrity of unfamiliar FIR in AD in challenging conditions (i.e. one fixation per face) yet without the confounding factors of explicit tasks. If AD patients’ ability to discriminate/match unfamiliar faces for their identity is truly impaired, we hypothesize a reduction of their FIR EEG response amplitude compared with cognitively unimpaired matched individuals. Moreover, in order to assess the impact of clinical stage on FIR ability, we evaluated how this EEG response differs according to the patients’ general cognitive deficits severity (i.e. classified as either MCI or dementia).

**Figure 1:**
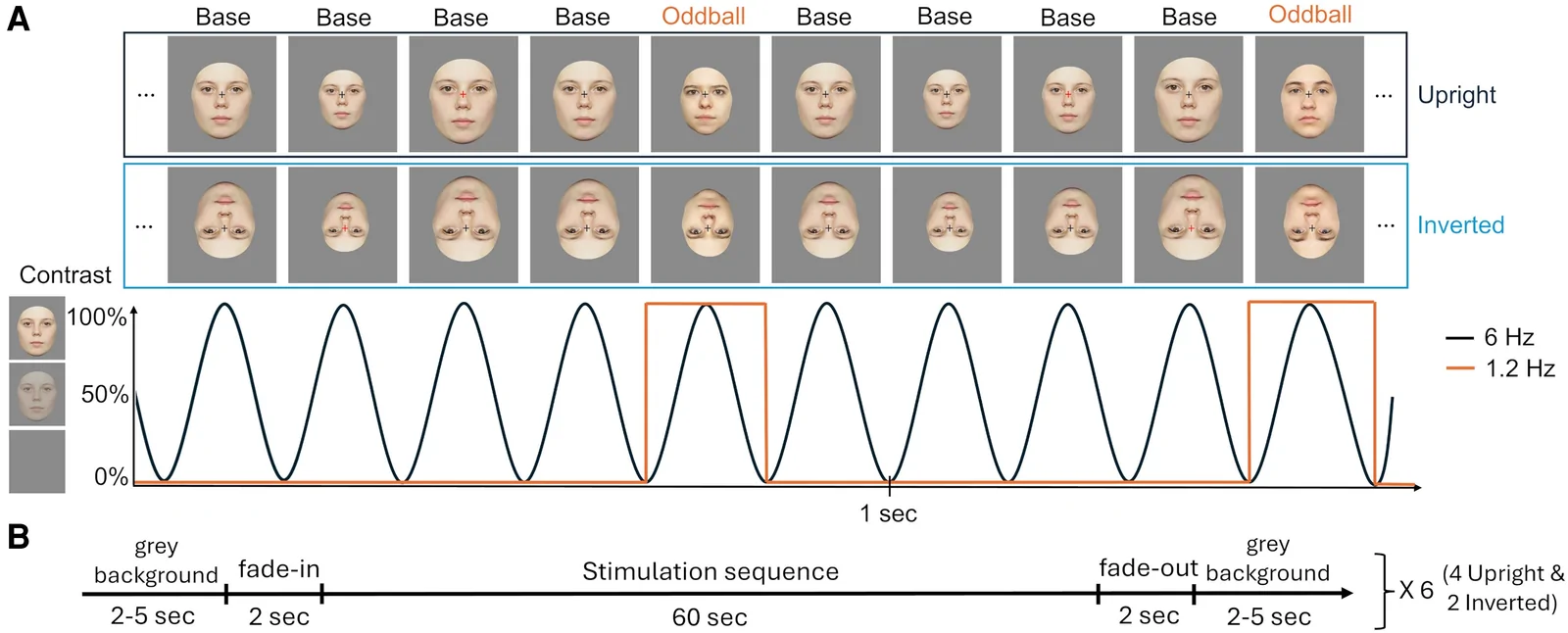
**FPVS-EEG paradigm (A) schematic representation of the paradigm.** The same unfamiliar face identity is presented at a frequency of 6 Hz (Base) with a different identity being presented every five stimuli (Oddball) (Images with permission). As represented at the bottom of the figure, stimuli are presented through sinusoidal contrast modulation, with full contrast being reached at half presentation time. Faces are presented either upright or upside down. A blue cross presented in the middle of the screen randomly changes colour (from blue to red), participant must press a button each time the colour changes. (**B**) A stimulation sequence starts with 2 to 5 s of grey background and a 2 s stimulus fade-in, then the stimulation sequence lasts for 60 s and ends with 2 s of fade-out and 2 to 5 s of grey background. Each participant completed 6 sequences.

## Materials and methods

### Participants

We tested 60 participants: 30 Ad patients (mean age: 73.3 ± 7; 19 females) and 30 healthy aged control participants (CTR, mean age: 71.8 ± 6; 20 females) matched for gender, age, and education level (Table 1). Sample size was determined based on previous studies comparing groups with this paradigm (e.g.,^42,47,49^ Ns < 30) and EEG studies of AD (e.g. *N* = 25^50^; *N* = 20^43^).

**Table 1:** Demographic data of Alzheimer’s disease patients and control subjects

	Alzheimer’s disease patients *n* = 30			Control subjects *n* = 30			*P*-value*
Age (years)	Min	Max	Mean (±SD)	Min	Max	Mean (±SD)	*P* = 0.38
	59	84	73.3 ± 7	59	85	71.8 ± 6	
Education level (Nb participants)							
Primary education	5			2			*P* = 0.52
Secondary education	3			2			
Post-secondary education	22			26			
Percentage of females (%)	63.33			66.67			*P* = 1.0
MMSE [Mean (±SD)]	23.60 ± 3.14			28.9 ± 0.83			*P* < 0.0001
Memory composite *z*-score [Mean (±SD)]	−4.28 ± 1.92			0.16 ± 0.63			*P* < 0.0001
Executive composite *z*-score [Mean (±SD)]	−1.38 ± 1.44			0.23 ± 0.33			*P* < 0.0001
Language composite *z*-score [Mean (±SD)]	−1.48 ± 1.04			0.02 ± 0.62			*P* < 0.0001
Visuo-spatial composite *z*-score [Mean (±SD)]	−1.49 ± 1.68			0.25 ± 0.46			*P* < 0.0001
Global cognitive composite *z*-score [Mean (±SD)]	−2.16 ± 1.08			0.19 ± 0.31			*P* < 0.0001
CSF amyloid rate (pg/mL) [Mean (±SD)]	346.4 ± 138.4			/			/
CSF phospho-tau rate (pg/mL) [Mean (±SD)]	95.79 ± 42.60			/			/
CSF total-tau rate (pg/mL) [Mean (±SD)]	611.7 ± 271.3			/			/

^*^ *P*-values were calculated using Fisher’s exact test for the education level, Chi-squared test for gender (χ^2^ = 0), Student *t*-test for age (*t*_58_ = 0.88) and global cognitive composite score (*t*_50_ = 10.9), and Mann-Whitney Wilcoxon tests for MMSE (*W* = 709), memory composite score (*W* = 788), executive composite score (*W* = 745), language composite score (*W* = 666) and visuo-spatial composite score (*W* = 643).

Patients were French-speaking Belgians recruited at the memory clinic of Saint-Luc University Hospital in Brussels. They had a biological diagnosis of AD^51^ as defined by cerebrospinal fluid (CSF; *n* = 28) amyloid-b42 concentration <437 pg/mL^52^ or a total tau/amyloid-b42 ratio >1.2 or a Centiloid value >26^53^ on amyloid-PET imaging (using [^18^F]-Flutemetamol, *n* = 2). CSF biomarkers (amyloid-β42, total tau) were measured using Lumipulse automated immunoassays. Cognitive impairment was documented through a comprehensive neuropsychological assessment evaluating four cognitive domains: episodic memory (Free and Cued Selective Reminding Test, French version),^54^ language (Lexis Naming Test, the Category Fluency Test for animals, and the Letter Fluency Test for the letter ‘P’),^55^ executive functions [Trail Making Test part A and B, Luria’s Graphic Sequences (adaptations in French, unpublished)], and visuospatial abilities (Clock Drawing Test and the Praxis part of the CERAD battery).^56,57^ A composite score (*z*-score) was computed based on three measures for each of these cognitive domains (Table 1).^58^ Participants also completed the Mini-Mental State Examination (MMSE).^59^ Patients were characterized by impaired memory performance on neuropsychological tests (memory *z*-score <−1.5), either in isolation or in combination with impaired performance in another cognitive domain (language, executive, visuospatial).

Control participants were recruited through mailbox announcements and advertisements in the hospital’s vicinity. Inclusion criteria for this group were a MMSE score ≥27/30 and cognitive performance in the normal range on neuropsychological assessment (all *z*-scores >−1.5).

For both groups, exclusion criteria were suspected non-AD neurodegenerative disorders (as supported by CSF Aβ42 concentration ≥437 pg/mL, a CSF total tau/amyloid-β42 ratio ≤1.2, or a Centiloid value <26, as well as clinical and/or imaging evidence), focal brain lesions, psychiatric diseases, alcohol or drug abuse.

All participants gave written consent before starting the study and the protocol was approved by the ethical committee Eudra-CT 2018-003473-94 of UCLouvain. They received financial compensation for their participation.

In a second step, and in order to investigate the influence of clinical stage on the FIR EEG measure, the AD patient group was divided into two subgroups based on patients’ cognitive deficits severity and degree of functional impact on daily activities. Patients with an MMSE score ≥24 and at least one cognitive *z*-score below −1.5 were considered as MCI due to AD patients (*n* = 17),^60^ while patients with an MMSE score <24 and at least two cognitive *z*-scores below −1.5 (including a memory *z*-score <−1.5) were classified as dementia due to AD patients (*n* = 13).^61^ These subgroups were matched for gender, age and education level (Table 2). The correspondence between the cognitive diagnosis (MCI or dementia) and the degree of functional impact on daily activities (minimal for MCI versus substantial interference for dementia) was verified by neurologists (B.H., A.I., L.C.) and neuropsychologists (L.Q., L.H.). Any discordances were resolved through consensus.

**Table 2:** Demographic data of MCI due to AD patients, dementia due to AD patients and control subjects

	Control subjects *n* = 30	Mild Cognitive Impairment due to Alzheimer’s disease patients *n* = 17	Dementia due to Alzheimer’s disease patients *n* = 13	*P*-value
Age [Mean (±SD)]	71.8 ± 7	72.6 ± 7	74.1 ± 7	*P* = 0.58
Education level (Nb participants)				
Primary education	2	2	3	*P* = 0.38
Secondary education	2	1	2	
Post-secondary education	26	14	8	
Percentage of females (%)	66.67	64.71	61.54	*P* = 0.95
MMSE [Mean (±SD)]	28.9 ± 0.83	25.8 ± 1.95*	20.8 ± 1.83*,**	*P* < 0.0001
Memory composite *z*-score [Mean (±SD)]	0.16 ± 0.63	−3.76 ± 1.96*	−5.32 ± 1.51*	*P* < 0.0001
Executive composite *z*-score [Mean (±SD)]	0.23 ± 0.33	−0.81 ± 1.13*	−2.52 ± 1.25*,**	*P* < 0.0001
Language composite *z*-score [Mean (±SD)]	0.02 ± 0.62	−0.93 ± 0.62*	−2.28 ± 1.07*,**	*P* < 0.0001
Visuo-spatial composite *z*-score [Mean (±SD)]	0.25 ± 0.46	−0.87 ± 1.17*	−2.54 ± 1.84*	*P* < 0.0001
Global cognitive composite *z*-score [Mean (±SD)]	0.19 ± 0.31	−1.59 ± 0.66*	−3.14 ± 0.93*,*	*P* < 0.0001
CSF amyloid rate (pg/mL) [Mean (±SD)]	/	332.6 ± 132.4	364.7 ± 149.9	*P* = 0.55
CSF phospho-tau rate (pg/mL) [Mean (±SD)]	/	87.75 ± 35.14	106.5 ± 50.51	*P* = 0.26
CSF total-tau rate (pg/mL) [Mean (±SD)]	/	571.9 ± 227.7	664.7 ± 323.5	*P* = 0.38

*P*-values reported in the last column were calculated using Fisher’s exact test for the education level, Chi-squared test for gender (*χ*^2^ = 0.11), ANOVA for age (*F*_2,57_ = 0.55) and global cognitive composite score (*F*_2,49_ = 123), Kruskal-Wallis tests for MMSE (H_2_ = 40.9), memory composite score (H_2_ = 38.9), executive composite score (H_2_ = 33.8), language composite score (H_2_ = 35.2) and visuo-spatial composite score (H_2_ = 27.5) and independent samples *t*-test for CSF amyloid (*t*_26_ = 0.60) phospho-tau (*t*_26_ = 1.16) and total-tau (*t*_26_ = 0.89) rates. *Post hoc* pairwise comparisons were performed using *t*-tests with Tukey correction. Results of these *post hoc* tests are reported with asterisks.

^*^Significantly different from Controls (*P* < 0.05).

^**^Significantly different from MCI due to AD patients (*P* < 0.05).

### Benton facial recognition test

Participants were tested with the BFRT-c,^62^ a widely used, challenging (no ceiling effect) and validated measure of unfamiliar FIR.^63,64^ This test requires to match greyscale photographs of faces presented on a computer screen based on their identity. In the first part of the test (6 trials), the face identity at the top of the screen corresponds to one of 6 face identities presented below. In the second part (16 trials), participants have to select among the six faces, the three faces that correspond to the target face, with changes of lighting and head orientation. Accuracy was calculated by counting one point per correct response, with a maximum score of 54 (6 + 3 × 16). Scores were corrected for age using normative adjustment procedures.^65^

### EEG acquisition

High-density 68-channel EEG was recorded with an ActiveTwo system (BioSemi, Amsterdam, The Netherlands), with a standard (10–20) 64-channel montage completed by 4 additional occipito-temporal electrodes (left: PO9, I1; right: I2, PO10).^50,66,67^ Offsets of the electrodes, referenced to the common mode sense (CMS), were held below 30 mV. Three flat-type active electrodes were used to record electrooculogram (EOG): one above the participant’s right eye and two laterals to the external canthi of the two eyes. EEG and EOG were digitized at 512 Hz.

### Stimuli

The stimulus set was the same as used in previous studies,^39,49,68^ composed of 25 male faces and 25 female faces, unknown to the participant, with a neutral expression, cropped to remove any external feature (e.g. hair, ears) and displayed on a grey background (Fig. 1). The final stimuli had a height of 290 ± 9 pixels and a width of 188 ± 12 pixels. Shown at a distance of 80 centimetres from the computer monitor, the stimuli had a visual angle of ∼5 × 4 degrees. Inverted faces were created by vertically flipping the stimuli around the horizontal axis.

### Experimental procedures

The experiment was run in a quiet and low-lit room. Stimuli were presented on a 120 Hz refresh rate VIEWPixx screen. The paradigm was the same as in^39^: the same face identity appears at a frequency of 6 Hz (i.e. 6 images per second) with different identities (selected randomly from the 24 faces remaining in the set) appearing every 5 images, i.e. at 1.2 Hz (6/5) (Fig. 1A). As presented in Fig. 1B, each sequence began with a 2 to 5-second grey background with the fixation cross followed by a 2-second fade-in during which stimulus contrast gradually increased from zero to full contrast in order to reduce expectation and behavioural reaction (e.g. blinks) to sudden stimulus onset. Then, stimuli were presented for 60 s, the sequence ending with 2 s of fade-out (Fig. 1B). Stimuli were presented through sinusoidal contrast modulation,^69,70^ with each presentation beginning with a uniform grey background from which a face appeared as its contrast increased. Each presentation cycle lasted 166 ms (i.e. 1000 ms/6), full contrast was reached at 83 ms and then decreased at the same rate (Fig. 1A). To minimize low-level effects, stimulus size varies at every presentation cycle between 74% and 120%. Two sequences were run with the female face stimulus set and two with male faces. To further control for low-level effects, two additional sequences were run with stimuli presented upside down (inverted face condition). In total, participants completed 6 sequences of about 66 s. Sequence order was randomized for each participant. As in other FPVS-EEG studies, including with AD populations,^43,50^ participants performed an orthogonal task of pressing on a response box button when the central cross briefly changed colour (1 s, blue to red; 10 random changes by sequence). Task performance was close to ceiling for both groups (AD: 97.5% ± 4; CTR: 98.4% ± 2) (*U* = 377, *P* = 0.26), and mean correct Response Times (RT) (AD: 510 ± 128 ms; CTR: 488 ± 125 ms) did not differ between groups (*U* = 515.5, *P* = 0.34). No significant differences were found between the three subgroups dementia due to AD: 96% ± 5; MCI due to AD: 99% ± 2, H_2_ = 3.12, *P* = 0.21; RT: (533 ± 175 ms versus 491 ± 78 ms, H_2_ = 1.03, *P* = 0.60).

### EEG analysis

EEG analyses were performed in *Letswave 6*, an open-source toolbox (https://www.letswave.org/) running over MATLAB R2021a (MathWorks, USA). Data processing steps are similar to previous studies.^39,49,68,71^ First, individual data files were band-pass filtered (Butterworth 0.1–100 Hz), and a Fast Fourier Transform (FFT) multi-notch filter with a width of 0.5 Hz was applied to remove electrical noise at 50 Hz frequency and 2 harmonics (100, 150 Hz). Following downsampling to 256 Hz to reduce computational load and storage space, the data were segmented by sequence (−2 s to +2 s; 64 s). Eyeblink correction was applied on the data of participants who blinked more than 0.2 times/sec on average during the sequence^72^ with independent component analysis (ICA), removing one clear artefactual component (14 Ad patients, 11 controls). Individual channels with artefacts (0–6 channels per participant; 1.52% of channels on average for AD patients and 0.93% for control participants) were interpolated with a linear combination of three neighbouring channels. All EEG channels were re-referenced to a common average reference. Each sequence was then re-segmented to an integer number of 1.2 Hz cycles, excluding fade-in and fade-out stimulation portions. The resulting 60-second epochs were averaged in the time domain separately for each condition for each participant to increase SNR. Fast Fourier Transform was applied, and amplitude spectra extracted with a high frequency resolution of 0.0167 Hz (1/60 s; 7680 frequency bins).

The main Regions Of Interest (ROI) were defined based on previous studies^41,49^ and inspection of topographical maps for both groups. ROIs for the identity discrimination EEG response at 1.2 Hz and harmonics were the right and left occipito-temporal (right or left OT) regions (four contiguous channels in each hemisphere: P8/P7, P10/P9, PO8/PO7, PO10/PO9). For the general visual response (6 Hz and harmonics), the ROI was the middle occipital (MO) region (six contiguous channels: I1, Iz, I2, O1, Oz, O2). To identify significant responses at the frequency of interest and harmonics, *Z*-scores were computed by subtracting the mean baseline noise from the amplitude at the frequency bin of interest and dividing by the baseline standard deviation. *Z*-scores were calculated across the ROI and the average of all 68 electrodes on the FFT spectrum average across individuals for each face orientation and group. Baseline noise level was estimated as the average of twenty-two surrounding frequency bins, excluding immediately adjacent bins in case of spectral leakage (i.e. 10 frequency bins on each side, frequency range of 0.334 Hz). Responses were considered significant if the *Z*-score was above 1.64 (*P* < 0.05, one-tailed, i.e. signal > noise). To quantify the response, the EEG spectrum was segmented into successive chunks centred on the bin containing the identity discrimination frequency (1.2 Hz, 2.4 Hz, etc.) and chunks of significant harmonics were summed.^72,73^ Each chunk contained 25 bins (i.e. length of 0.4175 Hz). Then, two methods were used to quantify and visualize the response: (1) subtraction of the baseline activity from the signal (corrected amplitude in µV), (2) division of the signal by the baseline noise (SNR). Baseline EEG activity was estimated as the average of 24 surrounding bins, excluding the immediately adjacent bins. The local maximum and minimum amplitude bins were also excluded for spectra visualization purposes (i.e. avoiding including the bin containing signal for noise calculation in neighbouring bins), providing also 10 frequency bins on each side in total. A greater number of bins was used compared with the *Z*-score calculation in order to maintain a consistent effective baseline of 20 bins across all analyses.

We also quantified an inversion effect for each participant individually by subtracting the FFT spectrum for Inverted faces from the spectrum of Upright faces. Subsequent analyses were then performed on this difference FFT spectrum using the same procedures described above. To quantify the inversion effect, responses from the ROI of each hemisphere were pooled into a single ROI comprising eight channels.

### Statistical analysis

Statistical analyses were conducted in RStudio (v2024.12.0). Group differences in BFRT-c accuracy were assessed with a Welch *t*-test, RT with Mann-Whitney-Wilcoxon tests, and subgroup proportions below the referential threshold with Fisher's exact test.

EEG response amplitudes were analysed with linear mixed-effects models (LMEs; lmerTest package). For the FIR response, a model including *Face Orientation*, *Group*, *Hemisphere*, their three-way interaction, and random slopes for *Face Orientation* and *Hemisphere* by participant was fitted. This random-effects structure was retained because it provides a better balance between model fit and complexity, as indicated by lower AIC and BIC values compared with models without random slopes. For the general base-frequency response (6 Hz and harmonics), analyses were restricted to mid-occipital electrodes; a model with *Face Orientation*, *Group*, and their interaction was fitted with random intercepts only (random slopes excluded due to non-convergence). In both models, the Control group and Upright condition served as references. Fixed effects were evaluated with Type III *F*-tests (Satterthwaite's method), and significant effects followed up with Tukey-corrected pairwise contrasts (emmeans).

The face inversion effect (upright minus inverted FIR amplitude) was compared between groups using a one-way ANOVA with Tukey-corrected *post hoc* contrasts.

Individual FIR responses were correlated with neuropsychological scores, BFRT-c performance, and tau-PET SUVr (Supplementary material 3) using Spearman correlations, with FDR correction for multiple comparisons (Supplementary material 4 and 5 and Supplementary Fig. 3).

## Results

### Overall comparison: AD patients versus controls

#### Benton facial recognition test (BFRT-c)

While all 30 control participants completed the BFRT-c, the BFRT-c was stopped before the end for 11 Ad patients, as they were unable to complete the test, complaining about its difficulty and duration. In particular, they had difficulties in remembering which face identities were already selected during the second part of the test requiring to select several items in turn.

Mean accuracy was 38.6 ± 4.7 for AD patients and 43.9 ± 2.4 for CTR (*t*_24.1_ = 4.54, *P* < 0.001, *d* = 1.53) (Fig. 2A). Twelve AD patients out of 19 performed below the established threshold of 41/54,^65^ against only two control participants. There was also a significant difference between groups for the total time taken to complete the test (AD: 374 ± 110 s; CTR: 264 ± 82.7 s; *U* = 444, *P* = 0.001, *r* = 0.56) (Fig. 2B).

**Figure 2:**
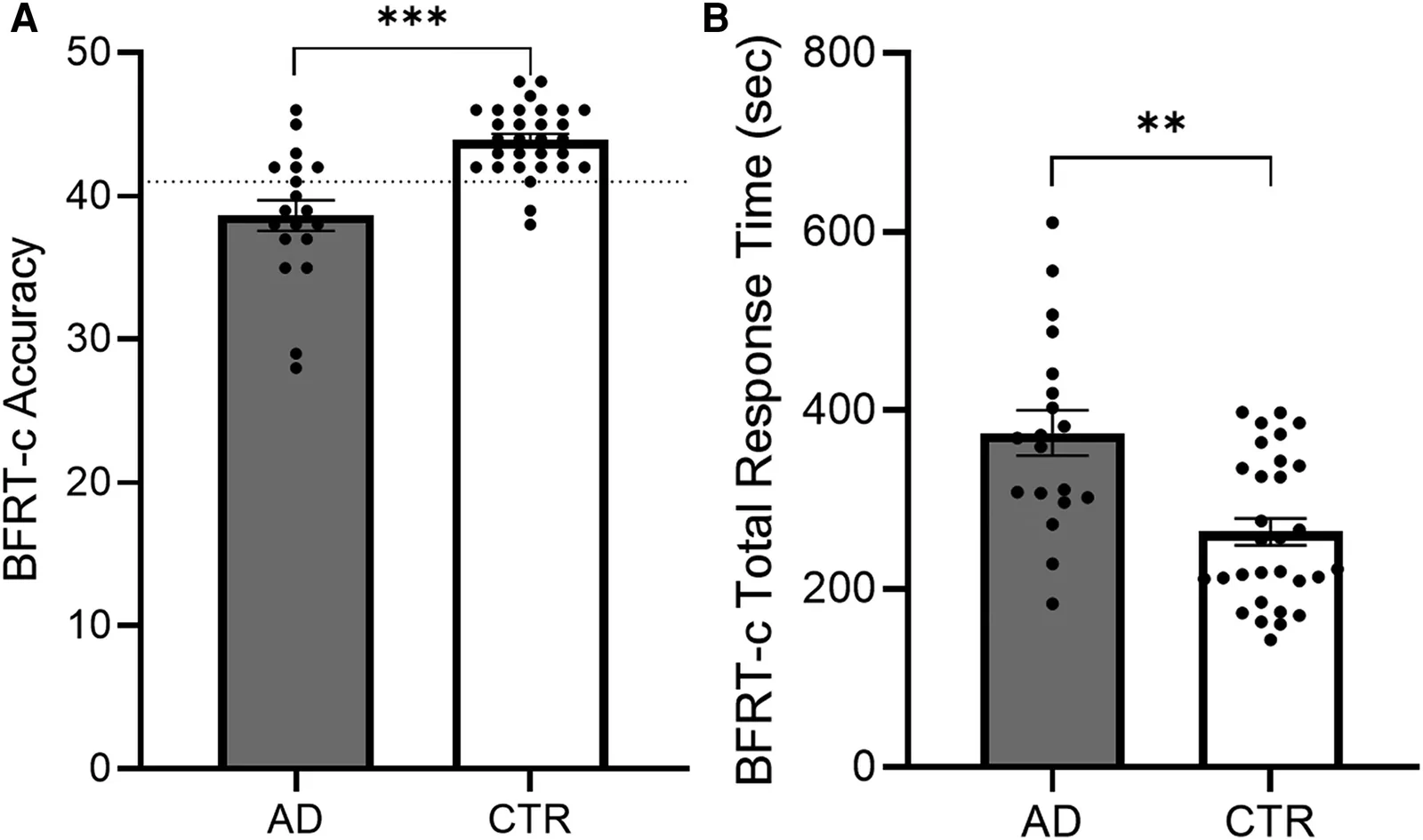
**Benton facial recognition test (BFRT-c) results.** (**A**) BFRT-c Accuracy (scores/54) and (**B**) BFRT-c total RT, for both groups [19 Alzheimer’s patients (AD) and 30 controls (CTR)]. Each dot represents one participant, and bars represent standard errors of the mean. For the BFRT-c accuracy, the black dotted line represents the accuracy score threshold (41/54). ***: *t*_24.1_ = 4.54, *P* < 0.001; **: *U* = 444, *P* = 0.001 (significance value are taken from Welch *t*-test for accuracy and from Mann-Whitney-Wilcoxon tests for the total response time presented in full in the result section).

#### General visual EEG response

A significant amplitude response was observed for both groups and face orientations at the general 6 Hz visual stimulation frequency up to nine consecutive harmonics in the AD patients group for the inverted face condition (i.e. 6 Hz, 12 Hz, until 54 Hz, *Z* > 1.64, *P* < 0.05) at the middle occipital ROI and up to eight consecutive harmonics (from 6 to 48 Hz) in the other orientation condition and groups. Quantification of the EEG response was therefore computed as the sum of nine harmonics. Scalp map topographies of the baseline-corrected amplitude at the general visual base frequency were highly similar between groups (Supplementary Fig. 2). Statistical analyses showed no significant effect of *Group* (*F*_1,58_ = 0.47, *P* = 0.49, *ƞ*^2^ = 0.008), a significant main effect of *Face Orientation* (*F*_1,58_ = 5.3, *P* = 0.02, *ƞ*^2^ = 0.08; Upright: 2.11 ± 1.14 µV > Inverted: 1.96 ± 1.12 µV) and no significant interaction (*F*_1,58_ = 0.39, *P* = 0.53, *ƞ*^2^ = 0.007).

#### Face identity recognition EEG response

Clear EEG response peaks were observed for both groups at the FIR frequency (i.e. 1.2 Hz) and harmonics (2.4 Hz, 3.6 Hz, …) for the upright faces condition at bilateral occipito-temporal electrode sites (Fig. 3A). In contrast, 1.2 Hz responses and harmonics were extremely weak or absent for inverted faces for both groups (Fig. 3).

**Figure 3:**
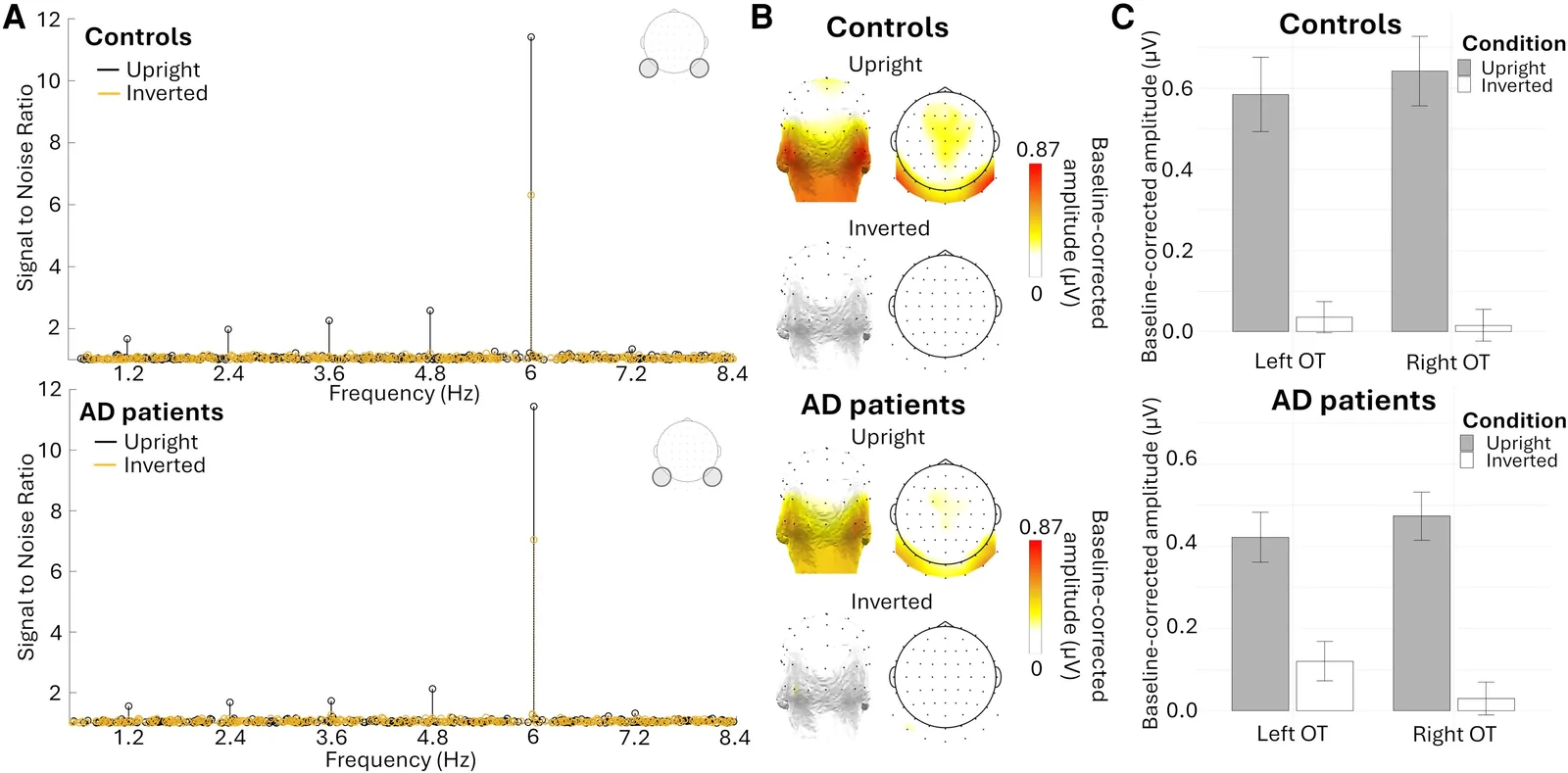
**Face identity recognition EEG response.** (A) Frequency spectrum of signal to noise ratio (SNR) quantified at bilateral occipito-temporal region in black for upright faces and in orange for inverted faces, above for controls (*N* = 30) and below for Alzheimer’s disease (AD) patients (*N* = 30). Each data point represents the SNR at the corresponding frequency. High EEG amplitude peaks are present at general visual frequency (6 Hz) for both groups and both Face orientations, and at face identity discrimination frequency (1.2 Hz and harmonics) for both groups but only for upright faces. (**B**) Scalp map topographies (3D maps on the left and 2D maps on the right) of the baseline-corrected amplitude response at face identity discrimination frequency (1.2 Hz and harmonics) summed across five harmonics. (**C**) Bar plots representing baseline-corrected amplitude of the face identity discrimination response for Upright faces (grey colour) and for Inverted faces (white) quantified in left OT (on the left) and right OT (on the right). Bars represent standard error of the mean.

For upright faces, on the average of all 68 electrodes, the baseline-corrected amplitude was significant until the 5th harmonics of 1.2 Hz for both groups, excluding the 6 Hz frequency (i.e.1.2 Hz, 2.4 Hz, 3.6 Hz, 4.8 and 7.2 Hz; *Z* > 1.64, *P* < 0.05). Therefore, EEG amplitude was summed across five harmonics in all subsequent analyses. For inverted faces, no significant *Z*-scores were found at any 1.2 Hz harmonics on the average of all 68 electrodes (all *Z* < 1.64, *P* > 0.05).

Scalp map topographies of the baseline-corrected amplitude of the FIR EEG response summed across harmonics showed a maximal amplitude at bilateral occipito-temporal sites for both groups (Fig. 3), with a highly significant correlation between groups’ mean response amplitude across electrodes (*r*_s_ = 0.89, *P* < 0.001). Numerically, in the upright condition, the FIR response was smaller in the AD group than the control group in both ROIs (28% reduction in the left hemisphere; 26% reduction in the right hemisphere; Fig. 3).

Data from one control participant was considered an extreme outlier and excluded from statistical analyses since it lays beyond Q3 + 3 × IQR (i.e. 0.71 + 1.52 = 2.23 < 2.24 µV). Statistical analyses computed on corrected amplitude response at the FIR frequency (1.2 Hz and harmonics) showed a significant effect of *Face Orientation* (*F*_1,57.17_ = 114.81, *P* < 0.0001, *ƞ*^2^ = 0.67; upright>inverted). No significant effect of *Group* (*F*_1,56.37_ = 0.77, *P* = 0.38, *ƞ*^2^ = 0.01) or *Hemisphere* was found (*F*_1,58.03_ = 0.05, *P* = 0.83, *ƞ*^2^ = 0.0008). There was a significant interaction between *Group* and *Face orientation* (*F*_1,57.17_ = 5.15, *P* = 0.03, *ƞ*^2^ = 0.08) due to a non-significant (*b* = −0.05, SE = 0.05, *P* = 0.30, 95% CI [−0.15; 0.05]) higher amplitude response for AD patients (0.08 ± 0.24 µV) compared with controls (0.03 ± 0.21 µV) for the *Inverted* face condition and the opposite pattern for the *Upright* condition (CTR: 0.59 ± 0.44 > AD: 0.45 ± 0.32, *b* = 0.15, SE = 0.09, *P* = 0.10, 95% CI [−0.03; 0.33]). Importantly, a strong inversion effect was found in both groups (*P* < 0.001). In addition, a significant interaction was found between *Hemisphere* and *Face Orientation* (*F*_1,57.86_ = 5.58, *P* = 0.02, *ƞ*^2^ = 0.09) as amplitude was non-significantly higher in the right OT region than in the left OT region for the Upright face condition (*b* = −0.07, SE = 0.04, *P* = 0.09) and the inverse pattern was found in the Inverted face condition (*b* = 0.06, SE = 0.04, *P* = 0.16). However, EEG amplitude was highly significantly larger for upright than inverted faces in both hemispheres (Right OT: *b* = 0.54, SE = 0.05, *P* < 0.0001; Left OT: *b* = 0.41, SE = 0.05, *P* < 0.0001). No significant interaction was found between *Hemisphere* and *Group* (*F*_1,58.03_ = 0.81, *P* = 0.37, *ƞ*^2^ = 0.01) or between the three factors (*F*_1,57.86_ = 0.11, *P* = 0.74, *ƞ*^2^ = 0.002).

In summary, while there was a clear numerical advantage for controls over AD patients in the EEG FIR response for upright faces (i.e. about 27% decrease of amplitude for patients for the two hemispheres combined), suggesting a reduced ability to visually discriminate/match face identities across image (size) changes in AD, this difference was not close to significance. While this lack of effect could be due to insufficient statistical power to detect a modest difference, we explored the alternative possibility of heterogeneity within the AD sample by examining whether separating the AD group according to cognitive deficits severity would reveal stage-dependent differences obscured in the full-group analysis.

### MCI due to AD versus dementia due to AD

In the following analysis, AD patients were divided into two sub-groups (i.e. MCI due to AD or dementia due to AD) based on their cognitive deficit level (see Materials and methods section).

BFRT-c analyses performed with each sub-group separately are presented in Supplementary material 1 (Supplementary Fig. 1).

#### Face identity recognition EEG response

While the FIR response was similar between controls (mean ± SD: 0.59 ± 0.44 µV) and MCI due to AD patients (0.55 ± 0.28 µV), it was reduced by 48% in the dementia due to AD group compared with CTR (0.32 ± 0.23 µV) (Fig. 4). In the Inverted faces condition, EEG amplitude was similarly low across sub-groups (Dementia due to AD: 0.09 ± 0.20 µV, MCI due to AD: 0.06 ± 0.20 µV, CTR: 0.03 ± 0.18 µV) (Figs 4 and 5).

**Figure 4:**
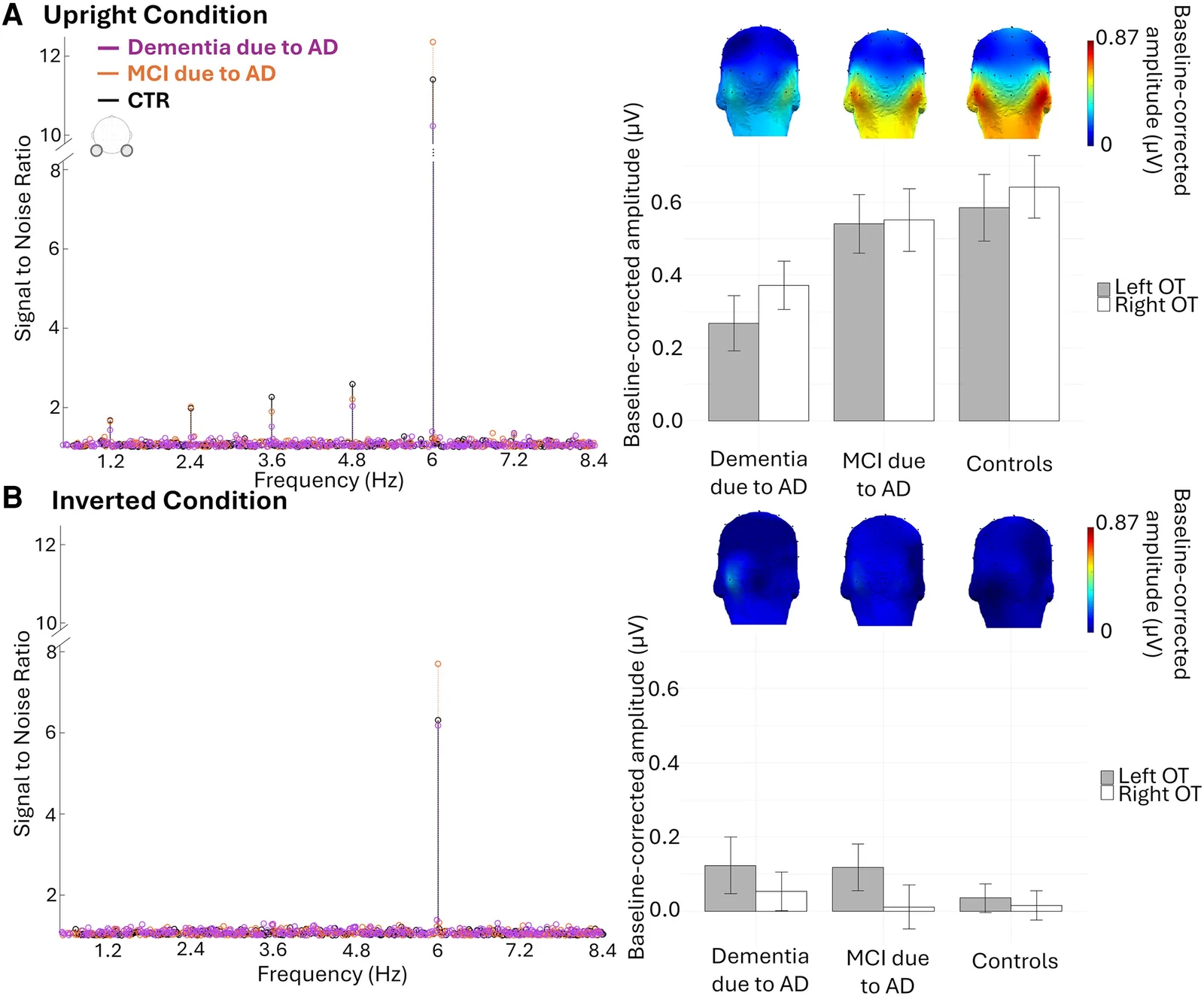
**Face identity recognition (FIR) EEG response by subgroups.** On the left are presented frequency spectra of Signal to Noise Ratio (SNR) quantified at bilateral occipito-temporal region for dementia due to Alzheimer’s disease (AD) patients (*N* = 13), for MCI due to AD patients (*N* = 17) and for Controls (CTR, *N* = 30). Each data point represents the SNR at the corresponding frequency. On the right are presented bar plots representing baseline-corrected amplitude of the face identity discrimination response quantified in the left Occipito-Temporal (OT) region and in the right OT region. Scalp map topographies (3D maps) of the baseline-corrected amplitude response at face identity discrimination frequency (1.2 Hz and harmonics) summed across five harmonics are presented above. These graphs are presented for both (**A**) Upright faces and (**B**) Inverted faces. A reduction of FIR response for Upright faces is observable in the dementia due to AD patients group compared with the other two sub-groups. Bars represent the standard error of the mean.

**Figure 5:**
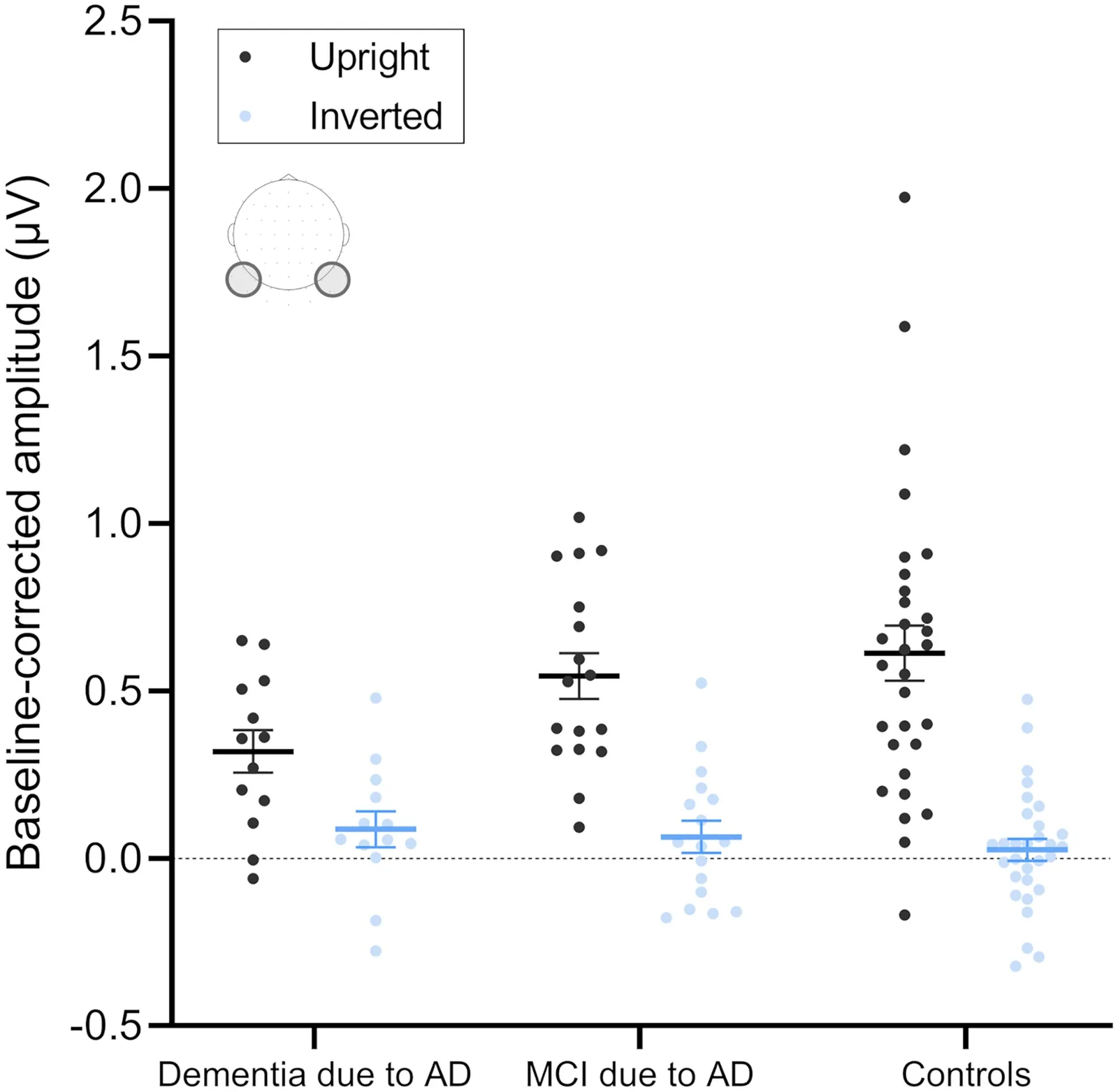
**Face identity recognition baseline-corrected amplitude quantified at bilateral occipito-temporal region for both face orientations for dementia due to Alzheimer’s disease (AD) patients (*N* = 13), MCI due to AD patients (*N* = 17), and controls (*N* = 30).** Mean and standard error are displayed, as well as each individual data point.

Statistical analyses computed on FIR response quantified in right OT and left OT ROIs revealed a significant effect of *Face Orientation* (*F*_1,55.94_ = 88.40, *P* < 0.0001, *ƞ*^2^ = 0.61) but no significant effect of *Hemisphere* (*F*_1,56.71_ = 0.0004, *P* = 0.98, *ƞ*^2^ < 0.00001) or *Sub-group* (*F*_2,55.29_ = 1.15, *P* = 0.32, *ƞ*^2^ = 0.04).

A significant interaction was found between *Face Orientation* and *Sub-Group* (*F*_2,55.97_ = 4.79, *P* = 0.01, *ƞ*^2^ = 0.15). *Post hoc* comparisons showed that this interaction was driven by lower FIR responses in the Upright condition for dementia due to AD patients compared with controls (*b* = 0.28, SE = 0.12, *P* = 0.048, 95% CI [0.002; 0.56]), not present for the Inverted condition (*b* = −0.06, SE = 0.06, *P* = 0.57, 95% CI [−0.21; 0.09]). No significant difference was found between the MCI due to AD group and controls in either condition (Upright: *b* = 0.05, SE = 0.11, *P* = 0.87, 95% CI [−0.20; 0.31]; Inverted: *b* = −0.04, SE = 0.06, *P* = 0.77, 95% CI [−0.18; 0.10]), nor between the two patient sub-groups (Upright: *b* = −0.23, SE = 0.13, *P* = 0.19, 95% CI [−0.53; 0.08]; Inverted: *b* = 0.02, SE = 0.07, *P* = 0.94, 95% CI [−0.14; 0.19]). All three sub-groups presented a significant inversion effect, which was smaller for dementia due to AD patients (*b* = 0.23, SE = 0.09, *P* = 0.01, 95% CI [0.05; 0.42]) than for the other two sub-groups (CTR: *b* = 0.57, SE = 0.06, *P* < 0.0001, 95% CI [0.45; 0.69]; MCI due to AD: *b* = 0.48, SE = 0.08, *P* < 0.0001, 95% CI [0.32; 0.64]). In addition, a significant interaction was found between *Hemisphere* and *Face Orientation* (*F*_1,56.51_ = 5.60, *P* = 0.02, *ƞ*^2^ = 0.09) due to a non-significantly higher amplitude in the right than the left hemisphere for upright faces (*b* = −0.07, SE = 0.04, *P* = 0.11, 95% CI [−0.15; 0.02]) and the inverse pattern for inverted faces (*b* = 0.07, SE = 0.04, *P* = 0.12, 95% CI [−0.02; 0.15]). No significant interaction between *Hemisphere* and *Sub-group* (*F*_2,56.75_ = 0.70, *P* = 0.50, *ƞ*^2^ = 0.02) or three-way interaction was found (*F*_2,56.56_ = 0.12, *P* = 0.88, *ƞ*^2^ = 0.004).

The inversion effect was then quantified for each participant individually. A significant effect of *Subgroup* on this inversion effect was found (*F*_2,57_ = 4.74, *P* = 0.01, *ƞ*^2^ = 0.14) with dementia due to AD patients having a significantly lower inversion effect (Mean ± SD: 0.24 ± 0.26 µV) than controls (0.60 ± 0.41 µV; *t*_57_ = 3.08, *P* = 0.009, *d* = 1.02) (Fig. 6). Inversion effect of the MCI due to AD patients (0.48 ± 0.30 µV) was similar to controls (*t*_57_ = 1.10, *P* = 0.52, *d* = 0.33). Finally, even if the mean inversion effect of dementia due to AD patients was reduced by half compared with MCI due to AD patients, this difference failed to reach statistical significance (*t*_57_ = 1.87, *P* = 0.16, *d* = 0.69) (Fig. 6).

**Figure 6:**
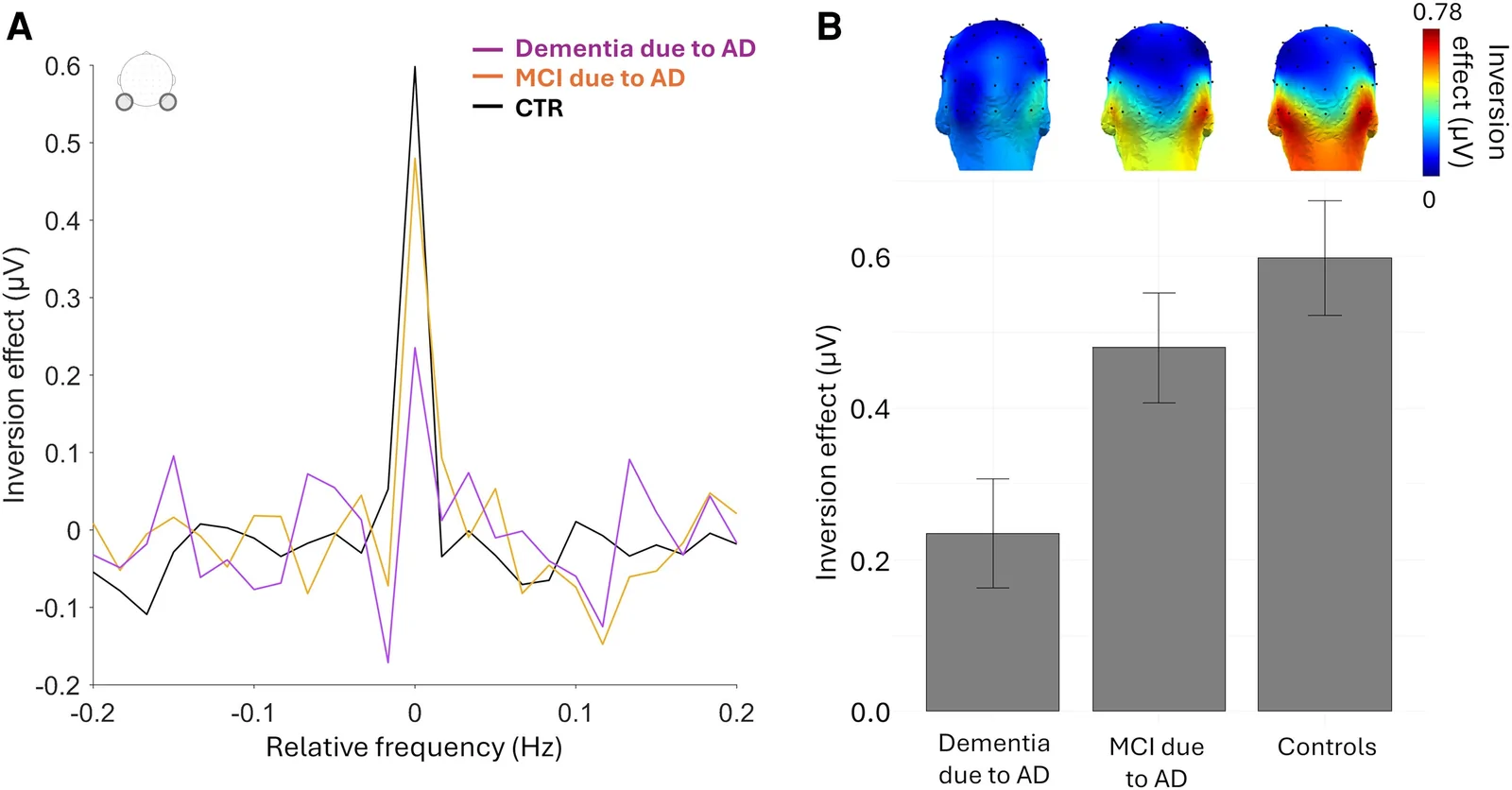
**Inversion effect.** (**A**) Baseline-corrected amplitude of the inversion effect at bilateral occipito-temporal ROI for dementia due to Alzheimer’s Disease (AD) patients in pink (*N* = 13), MCI due to AD patients in orange (*N* = 17) and controls (CTR) in black (*N* = 30). The spectrum is centred on the frequency bin of interest. Each data point represents the inversion effect at a specific frequency relative to the frequency of interest. (**B**) Barplots representing baseline-corrected amplitude of the inversion effect quantified at the bilateral OT ROI for each subgroup. 3-D scalp map topographies are presented above their respective subgroups. Bars represent the standard error of the mean.

At the base frequency (6 Hz and harmonics), similar amplitude responses were found between each subgroup (results presented in Supplementary Material 2 and Supplementary Fig. 2).

## Discussion

While FIR impairments are notorious in AD, they have been mainly investigated with explicit behavioural tasks involving episodic and/or semantic memory. However, human adults are also experts at unfamiliar/novel face recognition,^22^ i.e. the ability to discriminate different facial identities not encoded in memory and to generalize across different views of the same unfamiliar face identity. In this context, we used a highly sensitive and objective FPVS-EEG approach that has been recently successfully applied to assess old/new object image discrimination^43,44^ and word semantic categorization^50^ in dementia, here with a well-validated specific paradigm measuring unfamiliar FIR implicitly.^40^ Objectivity is ensured by quantifying the neural response exclusively at pre-defined frequency bins (1.2 Hz and harmonics) in the EEG frequency domain.^73^ Sensitivity stems from the large number of discriminated identities (288 trials) and high frequency resolution, minimizing broadband noise at frequencies of interest^37^, thus enabling detection of significant individual-level FIR responses in over 90% of neurotypical individuals within minutes.^40^

At the group level, we found a significant reduction of the face inversion effect in AD, but no statistically significant difference in the overall amplitude of the FIR response. One possible explanation for the absence of a significant group effect is the clinical heterogeneity of our patient sample, which included patients ranging from MCI to dementia. To explore this possibility, we conducted a complementary analysis, showing a significant reduction of the face inversion effect in AD patients at the dementia stage compared with controls, driven mainly by a reduced FIR response to upright faces, an effect absent in patients at the MCI stage.

The significant decrease of AD dementia patients’ FIR EEG response aligns with behavioural studies demonstrating unfamiliar FIR impairment in AD.^25-27^ Moreover, the smaller reduction in FIR EEG responses following face inversion in AD dementia is consistent with a diminished face inversion effect found in a behavioural face-matching task in patients with mild AD dementia.^21^ However, previous EEG studies with face stimuli in AD have not provided consistent outcomes (e.g. on the face-sensitive N170 component;^74-78^) and, most importantly, have not evaluated indexes of FIR (e.g. amplitude decreases with immediate repetition,^79^). Thus, our study reports, to our knowledge, the first implicit neural measure of reduced unfamiliar FIR function in dementia due to AD.

The attenuation of the EEG FIR response and the face inversion effect observed here suggest impairment in face perception in AD. Faces are processed differently from other types of visual stimuli and rely on specialized neuroanatomical substrates.^80-82^ Specifically, face identities are thought to be processed holistically, meaning that individual facial features are mandatorily integrated into a unified visual representation rather than as independent features.^83^ Thus, this altered FIR EEG response in AD may reflect a deficit in forming holistic visual representations of individual faces.^21^ This impairment might also be partly linked to AD patients’ difficulties in visuospatial organization^84^ or visual scanning pattern.^85,86^ Specifically, abnormal eye-movement patterns have been reported in AD during face viewing, with patients allocating less visual attention to key facial features such as the eyes and mouth compared with individuals with amnestic MCI and healthy controls.^86,87^ Future studies should aim to disentangle the cognitive mechanisms behind AD patients’ unfamiliar FIR deficit, for example, by investigating whether the visual perception impairment observed here is specific to faces or generalizes to other stimuli. Notably, we cannot rule out the possibility that (visual) working memory deficits may have partly contributed to their reduced FIR response. Indeed, since faces were presented sequentially, each face had to be briefly held in short-term visual memory before the next one appeared. However, and importantly, the reduction of FPVS-EEG FIR response found in the dementia due to AD group cannot be attributed to general reduction of neural signal amplitude, reduced attention, or diminished screen engagement since responses at the general visual presentation frequency and accuracy at the orthogonal task did not differ between groups. Finally, by bypassing task instructions, decision-making, and executive demands, the FPVS-EEG approach allows high specificity, making it unlikely that reduced FIR amplitudes reflect other general cognitive deficits associated with AD.

In contrast to these observations, no significant difference was found in the FIR response between MCI due to AD patients and controls. The preservation of FIR abilities in MCI patients contrasts with recently reported behavioural impairments in unfamiliar face identity matching and discrimination in prodromal AD,^10^ as well as the deficits in discriminating facial spatial configurations and changes in eye and mouth colour in patients with amnestic MCI.^32^ Similarly, in the present study, BFRT-c completers in the MCI group showed reduced accuracy and slower RT relative to controls, though the small sample size and lack of formal statistical analyses preclude definitive conclusions. There could be two main accounts of this discrepancy between EEG response and behavioural performance: either a lack of specificity of behavioural tasks that substantially involve general attentional, motivational, understanding and decision-making processes contributing to, or even fully accounting for, differences in performance between MCI patients and controls; or a lack of sensitivity of the FPVS paradigm. Supporting the former account, in the current study, more than one-third of AD patients did not successfully complete the task, especially when they had to select one by one three faces corresponding to the target face identity. In this case, the face identity items already selected have to be kept in (short-term) memory to avoid selecting some items twice (or even 3 times), something that proved particularly challenging for AD patients. Thus, even though this specific procedure of the BFRT-c constitutes a limitation that may not apply to other tests requiring only a two-alternative forced choice,^64^ behavioural tests with concurrently presented pictures of faces to explicitly discriminate/match appear to be challenging for amnestic MCI patients. Moreover, several studies have demonstrated the sensitivity of the FPVS paradigm used here to objectively defined FIR deficits (e.g. prosopagnosia).^45-47,49^ Indeed, FIR as measured in this paradigm is challenging (albeit not *behaviourally challenging*), since faces differ only in terms of internal features and with substantial variations of size, appearing at a rapid 6 Hz rate that prevent the reliance on compensatory mechanisms or alternative perceptual strategies that could mask deficits. Given these considerations, we consider that the preserved 1.2 Hz and harmonics response observed here reflects a genuine sparing of identity-specific perceptual processing.

Despite these arguments in favour of the sensitivity and specificity of the FPVS paradigm, we acknowledge that only one image per identity (across large variations of size) was used in our study, while the BFRT-c and the task used in Quenon *et al*.^10^ required participants to generalize across different views of the same face identity. Therefore, the present FPVS paradigm may not capture all dimensions of FIR, and some of which may be impaired as early as the MCI stage. Future studies could address this issue with FPVS-EEG paradigms that incorporate natural images with greater variability.^88,89^

Concerning the neuropathophysiological correlates of this FIR process, intracerebral EEG recordings with the FPVS paradigm as used here highlighted the VOTC in FIR response, in particular in the right hemisphere.^48^ Moreover, previous studies have shown AD patients’ neurodegeneration in regions implicated in face recognition, in particular the fusiform gyrus^90-93^ and a correlation between the fusiform gyrus atrophy and face processing abilities measured with explicit behavioural tasks.^94,95^ Abnormal face-selective activity in the middle fusiform gyrus and lateral inferior occipital cortex activation was found during face processing in MCI,^96,97^ even in the absence of behavioural impairment.^97^ Contrary to our expectations, however, no significant correlation was found between FIR responses and AD patients’ tau burden in VOTC regions here (Supplementary materials 4). This may be due to both our limited sample size and the influence of non-pathological sources of variability on EEG signals, such as differences in cortical folding, skull conductivity, or the anatomical organization of face-selective regions.

In conclusion, the present study provides the first comparison of unfamiliar FIR across MCI due to AD, dementia due to AD, and healthy elderly individuals. FPVS-EEG revealed preserved FIR neural response in MCI due to AD patients, but a significant reduction of this response in dementia due to AD patients. This dissociation suggests that FIR difficulties in MCI arise from other cognitive processes rather than impaired visual discrimination, whereas dementia involves a genuine perceptual deficit. Several clinical implications can be drawn from these findings. First, at the MCI stage, memory-based compensation strategies appear most appropriate, while at the dementia stage, visual perception strategies should also be incorporated. Second, the BFRT(-c) is not well suited to test specifically these abilities in patients with multiple major cognitive disorders, such as AD patients. In contrast, FPVS-EEG could provide a complementary tool to easily and rapidly measure face recognition ability in neurodegenerative populations and to disentangle specific cognitive processes underlying impaired behavioural performance. Together with previous research,^43,50^ our findings support the interest of the FPVS-EEG approach for the development of innovative cognitive assessment tools in clinical contexts. Incorporating such a specific, objective, and implicit measure of FIR abilities in AD patients’ clinical cognitive assessment could be particularly relevant and clinically useful, especially since this ability is essential for patients’ daily lives.

## Supplementary Material

fcag390_Supplementary_Data

## Data Availability

EEG and cognitive data as well as code used for statistical analyses are available in an OSF repository: https://osf.io/rdy9h/overview?view_only=2ec0f041f32c44c0a9274a7678ba62b3. Tau PET data are available for replication purposes or in the context of academic collaborations. Requests should be sent to bernard.hanseeuw@uclouvain.be.
