# Supplementary material for: Neural evidence for progressive decline of visual face recognition ability in Alzheimer’s disease
Source: Brain Commun. 2026 Oct 22;8(6):fcag390. doi: 10.1093/braincomms/fcag390 (PMC13725164; doi:10.1093/braincomms/fcag390)
Supplement: Supplementary file 1 [file fcag390_supplementary_data.docx]

**Supplementary Material**

1. **Benton Facial Recognition Test results by subgroup**

Out of the 19 AD patients who were able to complete the BFRT-c, 5 (out of 13) were classified as dementia due to AD patients, and 14 (out of 17) as MCI due to AD patients (significant difference between the two proportions: p=.02). Moreover, there is a gradient of accuracy decrease according to disease progression (MCI due to AD: 39.57 ± 4.31; dementia due to AD: 36.00 ± 5.15)(Supplementary Figure 1) and the proportion of AD dementia patients below the threshold of 41/54 was significantly higher (4 out of 5 ≈ 80%) than the proportion of CTR participants below 41/54 (2 out of 30 ≈ 6.7 %)(p=.001). Even if quantitatively lower than for dementia due to AD patients, the proportion of MCI patients below the threshold (8 out of 14 ≈ 57.1 %) was significantly higher than for CTR (p=.0006). Total RT was also increased for dementia due to AD patients (351 ± 79 sec) and MCI due to AD patients (383 ± 115) compared to CTR (264 ± 81) (Supplementary Figure 1).

Supplementary Figure 1**:** (A) Benton Facial Recognition Test (BFRT-c) Accuracy (scores/54) and (B) BFRT-c response times, for each group (Dementia due to Alzheimer’s Disease: N=5; Mild Cognitive Impairment due to AD: N=14; and Controls: N=30). Each dot represents one participant, and bars represent standard errors of the mean (SEM). For BFRT-c accuracy, the black dotted line represents the accuracy score threshold (41/54). Statistical comparisons of the proportion of individuals below the reference threshold between subgroups (Fisher's exact test) are not displayed on the graph, as the graph shows individual data points rather than proportions. The proportion of dementia due to AD patients below the threshold was significantly higher than the proportion of control participants below the threshold (p=.001). Even if quantitatively lower than for dementia due to AD patients, the proportion of Mild Cognitive Impairment patients below the threshold was also significantly higher than for CTR (p=.0006).

2. **General visual base EEG response by subgroup**

Similar amplitude responses at the base frequency were found between each subgroup, both for Upright faces (CTR: 2.04 ± 1.16 µV; MCI due to AD: 2.22 ± 1.16 µV; dementia due to AD: 2.15 ± 1.12 µV) and Inverted faces (CTR: 1.84 ± 0.99 µV; MCI due to AD: 2.18 ± 1.28 µV; dementia due to AD: 1.93 ± 1.24 µV). Statistical analyses confirm no significant main effect of *Subgroup* (F_2,57_=0.55, p=.58, ƞ^2^=0.02)(Supplementary Figure 2). A significant effect of *Face Orientation* was found (F_1,57_=4.26, p=.04, ƞ^2^=0.07) with a higher amplitude for the Upright condition than the Inverted condition. No significant interaction between *Subgroup* and *Face Orientation* was found (F_2,57_=0.51, p=.60, ƞ^2^=0.02).

Supplementary Figure 2: (A) Frequency spectrum of Signal to Noise Ratio (SNR) quantified at bilateral-occipito temporal region in pink for dementia due to Alzheimer’s disease (AD) patients (N=13), in orange for Mild Cognitive Impairment (MCI) due to Alzheimer’s disease patients (N=17) and in black for Controls (N=30), for Upright condition on top and for Inverted condition at the bottom. Each data point represents the SNR at the corresponding frequency. Clear peaks are observable for all subgroups at the general visual presentation frequency (6 Hz and harmonics). (B) Scalp map topographies of baseline-corrected amplitude response at general visual frequency for each subgroup and each condition. (C) Barplots representing baseline corrected amplitude response quantified across general visual frequency harmonics for each subgroup for the Upright condition in grey and the Inverted condition in white. Bars represent standard error of the mean.

3. **Tau-PET Imaging acquisition and analyses**

[18F]-MK-6240 (Lantheus Inc.) is an investigational drug studied as a second-generation cerebral tau tangles imaging agent. Ninety minutes after intravenous administration of [18F]-MK-6240 (target activity 185±5 MBq), a 30-min dynamic acquisition was performed on a Philips Vereos digital PET-CT. Images were reconstructed using manufacturer’s standard reconstruction algorithm (including attenuation, scatter, decay correction, and time-of-flight information). For all participants, tau-PET data were coregistrated with a 3D-T1 MRI using PetSurfer pipeline, a set of tools within FreeSurfer for end-to-end integrated MRI-PET analysis.^1^ Three ROIs were selected, based on regions of the face processing network: the fusiform gyrus, lateral-occipital cortex and inferior-temporal cortex. Tau SUVr was quantified in each hemisphere separately for each region, resulting in a total of six ROIs. Desikan-Killiany atlas ^2^ was used. SUVr values were extracted with the cerebellum gray matter as the reference region. Tau-PET data of three patients were excluded since the tau-PET scan was performed more than two years before the EEG-FPVS recording.

4. **Association between FIR EEG response and regional tau burden in face-processing brain regions**

Between-group comparisons of tau PET SUVR levels are reported in Supplementary Table 1.

**Supplementary Table 1: Tau PET SUVr across regions of interest and groups**

|  | *Control subjects*  *n=30* | *Mild Cognitive Impairment due to Alzheimer’s disease patients*  *n= 17* | *Dementia due to Alzheimer’s disease patients*  *n=13* | *Group effect* |
| --- | --- | --- | --- | --- |
| Tau SUVr (Mean (±SD)) |  |  |  |  |
| Left fusiform gyrus | 0.93 ± 0.12 | 2.30 ± 1.04* | 2.77 ± 1.23* | F_2,16.61_=25.1, p<.001 |
| Left inferior temporal cortex | 0.94 ± 0.12 | 2.50 ± 1.17* | 2.94 ± 1.55* | F_2,16.57_=22.3, p<.001 |
| Left lateral occipital cortex | 0.98 ± 0.12 | 1.88 ± 1.19* | 1.94 ± 0.90* | F_2,16.65_=10.6, p=.001 |
| Right fusiform gyrus | 0.93 ± 0.11 | 2.34 ± 1.04* | 3.18 ± 1.60* | F_2,16.56_=24.6, p<.001 |
| Right inferior temporal cortex | 0.95 ± 0.12 | 2.49 ± 1.10* | 3.15 ± 1.60* | F_2,16.58_=25.2, p<.001 |
| Right lateral occipital cortex | 1.00 ± 0.12 | 2.11 ± 1.32* | 2.47 ± 1.23* | F_2,16.57_=13.3, p<.001 |

For each region the main effect of group was assessed using Welch's ANOVA. Post hoc pairwise comparisons were performed using t-tests with Tukey correction. Asterisks indicate significant differences: * Significantly different from Controls (p < .05)

AD patients’ upright faces FIR EEG responses of each hemisphere were used to also examine correlations with regional tau burden. No significant correlation was found between patients’ FIR EEG responses and tau burden in any brain regions (all p’s >.43, Supplementary Figure 3). We can only note a negative correlation between FIR EEG amplitude in left OT and tau burden in the right lateral occipital cortex (r=-0.40, p=.04), but that did not survive FDR correction for multiple comparisons (p=.43, Supplementary Figure 3).

Supplementary Figure 3: (A) Matrix of correlation between normalized face identity recognition (FIR) EEG response at each hemisphere and tau PET SUVr in face processing brain regions of Alzheimer’s disease (AD) patients (N=27). (B) Matrix of correlation between normalized FIR EEG response and performance at different cognitive tests in the AD patients group (N=27) (C) and in the control group (N=25). For all three matrices, Spearman coefficients of correlation are displayed as well as FDR-corrected p-values. °= p<.10, *= p<.05. Colours represent the intensity and the direction of the correlation. A more intense colour represents a stronger correlation, while a lighter colour represents a weaker correlation. Negative correlations are represented in blue, while positive correlations are represented in red.

5. **Association between FIR EEG response and cognitive performances**

FIR EEG responses to Upright faces, quantified in bilateral OT, were used to examine correlations with cognitive scores. In the AD group, FIR responses did not significantly correlate with BFRT-c accuracy (r=0.18, p=.53) or response time (r=0.27, p=.38), nor in the control group (Accuracy: r=0.35, p=.14; response time: r=-0.11, p=.73)(Supplementary Figure 3). Regarding neuropsychological composite scores, in the AD group, a significant positive correlation was found with the visuo-spatial composite score (r = 0.49, p = .046). In controls, a correlation with executive functions composite score was observed (r = 0.49, p = .04), as well as with visuo-spatial composite score (r=0.48, p=.05)(Supplementary Figure 3).

**Supplementary References:**

1.	Greve, D. N. *et al.* Different partial volume correction methods lead to different conclusions: An 18F-FDG-PET study of aging. *NeuroImage* **132**, 334–343 (2016).

2.	Desikan, R. S. *et al.* An automated labeling system for subdividing the human cerebral cortex on MRI scans into gyral based regions of interest. *NeuroImage* **31**, 968–980 (2006).
